# The Impact of COVID-19 Vaccination on the Social Participation of Immunocompromised Persons – Results of a Multicenter Observational Study

**DOI:** 10.3389/fpubh.2022.877623

**Published:** 2022-06-10

**Authors:** Gloria Heesen, Dominik Schröder, Frank Müller, Eva Hummers, Frank Klawonn, Marie Mikuteit, Jacqueline Niewolik, Sandra Steffens, Anne Cossmann, Georg Behrens, Alexandra Dopfer-Jablonka, Stephanie Heinemann

**Affiliations:** ^1^Department of General Practice, University Medical Center, Göttingen, Germany; ^2^Department of Computer Science, Ostfalia University of Applied Sciences, Wolfenbuettel, Germany; ^3^Biostatistics Group, Helmholtz Centre for Infection Research, Braunschweig, Germany; ^4^Department of Rheumatology and Immunology, Hannover Medical School, Germany; ^5^German Center for Infection Research (DZIF), Partner Site Hannover-Braunschweig, Heidelberg, Germany

**Keywords:** social participation, observational study, COVID-19 vaccination, immunocompromised, pandemic (COVID-19)

## Abstract

Immunocompromised persons are at an increased risk for a severe SARS-CoV-2 infection and their safety behaviors may influence their social participation. Vaccinated persons have a lower incidence of infection and severe disease when infected compared to non-vaccinated persons. Therefore, their behavior may change and their social participation may increase after a complete vaccination. The aim of this study was to explore social participation of immunocompromised persons before and after complete COVID-19 vaccination. Between March and September 2021, 274 immunocompromised participants were recruited. Survey data were collected at baseline and follow-up from 194 participants including the Index for the Assessment of Health Impairments [IMET], Patient Health Questionnaire-4 [PHQ-4], subjective health status and quality of life. At baseline, participants were not yet completely vaccinated. Complete vaccination was achieved prior to the follow-up questionnaire. IMET scores decreased significantly at follow-up, indicating a higher social participation after complete vaccination. PHQ-4, subjective health status and quality of life did not differ between baseline and follow-up. There were no significant differences across sociodemographic factors. Significant PHQ-4 differences were observed regarding the population size of the participants' home community. Social participation of immunocompromised persons in our study increased after COVID-19 vaccination. Therefore, social participation should be explored further, especially with regards to the impact of vaccination on groups with a high health risk.

## Introduction

The global pandemic caused by SARS-CoV-2 is affecting daily life in various ways. Several “lockdowns” restricting social interactions to slow the spread of infection were adopted in Germany. This caused a fundamental change in daily social life e.g., social gatherings in spare time or at work were restricted by law. Before the release of the first vaccine in December 2020, the only proven protection against an infection were distance, masks, and hygiene measures.

Li et al. (1) proposed a positive association between the perceived severity and uncontrollability of COVID-19 with negative emotions and cautious behavior. It stands to reason that people at particularly high risk for a serious SARS-CoV-2 infection adopted risk-limiting behaviors that were more restrictive than the legal regulations implemented to protect the entire population (2). Furthermore, it is conceivable that such persons' family and friends learned to keep their distance because they feared infecting them unwillingly. Persons with immune dysfunction and concomitant immunosuppressive treatment for rheumatic and other autoimmune conditions are one group at particularly high risk (3).

Social participation is a broad concept and can be defined as involvement or being included in a community life situation (4, 5). In order to maintain and achieve personal autonomy and well-being, social participation is necessary (6, 7). Studies have already associated social participation with health outcomes (8). Immunocompromised persons are associated with a reduced social participation compared to healthy individuals (8). The pandemic situation presents an additional burden for social participation and mental health, even for healthy individuals (9–12).

Basic immunization is regarded a key effective protective measure against COVID-19, whereby the knowledge and recommendations about the number and timing of vaccinations is continuously changing throughout the pandemic. The definition of basic immunization against COVID-19 had to be repeatedly adapted to the current state of research in the course of the pandemic.

At the beginning of the survey (end of March 2021), a basic immunization was defined as 14 days after two doses of Comirnaty, Spikevax or Vaxzevria, or 14 days after one dose of COVID-19 Vaccine Janssen. According to the official recommendation in Germany, valid from December 2021, three vaccinations are needed for basic immunization of persons receiving an immunosuppressive medication such as Methotrexate or Cyclophosphamide and a booster vaccination after 6 months is also advised (13). At the start of the German vaccination campaign in December 2020, complete vaccination was thought to allow for relaxation of social restrictions even if the effectiveness of the vaccination for immunocompromised people was not entirely clear. Currently, breakthrough infections are occurring throughout Europe and beyond. Therefore, in contrast to original expectations, in German guidelines a complete relaxation of social restrictions is not recommended even after complete vaccination (14, 15).

More recently, the uncertainty and insecurity regarding individual vaccine effectiveness affects not only immunocompromised individuals, but all people due to viral variants of concern such as the omicron variant (16, 17).

The above-mentioned factors underline the need to understand the impact of vaccination upon social participation and quality of life. Therefore, this study aims to investigate if a complete COVID-19 vaccination influences social participation in a prospective, multicenter study with immunocompromised persons.

## Materials and Methods

### Research Design and Participants

The COVID-19 Contact Immune Study [CoCo study] is a prospective, longitudinal, observational study at two large university hospitals in Northern Germany that, besides others, included participants with immunosuppressive drug therapy. Recruitment took place between March 2021 and September 2021. Persons with an ongoing immunosuppressive medication who were 18 years or older and capable of giving consent were included in the Coco Immune Study. No further inclusion or exclusion criteria were applied.

The recruitment strategy consisted of newspaper advertisements, posters in vaccination centers, in university hospitals and in doctors' offices specialized in rheumatologic diseases. We set up a study telephone hotline and an e-mail address where interested participants could contact study personnel directly.

Due to the pandemic situation and the particularly vulnerable, immunosuppressed participant group, we conducted the study in a minimized-contact manner. Enrollment in the study and obtaining consent from participants could be done by video or phone call or in person, depending on participants' preference. Study materials were shipped by mail to the participants. Study materials were returned by mail. All participants were informed that that all possible preventive measures should be taken and all regulations should be observed. Further information can be gathered in the study protocol (18).

### Measures

#### Index for the Assessment of Health Impairments (IMET)

The primary outcome is the IMET [Index for the Assessment of Health Impairments], which is a self-administered questionnaire to measure social participation based on the International Classification of Functioning, Disability and Health (19). It was initially developed to assess participation and involvement in persons with different chronic diseases and validated in a large cohort. The main field of application is rehabilitation research. The IMET is unidimensional and consists of nine items with a 11 (0–10) level Likert scale where higher scores indicate lower social participation consistently across all items. The sum of all nine items can be used to determine the overall social participation with a high internal reliability (Cronbach's alpha 0.90). The IMET asks if the participants have any impairments at the moment. It does not measure the actual social behavior of the participants. The IMET was used by Mergel & Schützwohl to assess social participation before and after the COVID-19 lockdown in participants with a mental disorder and in the general population (20).

#### PHQ-4

The PHQ-4 [Patient Health Questionnaire-4] is a brief, validated, high reliability (α 0.85) measure of anxiety and depression symptoms (21). This scale consists of two subscales PHQ-2 [Patient Health Questionnaire-2] measuring depression symptoms and GAD-2 [Generalized Anxiety Disorder Scale-2] measuring anxiety symptoms, consisting of two four-point Likert-type items (0–3) for each subscale. It produces an overall psychological distress sum score ranging from 0–12, where higher scores indicate a worse psychological well-being. Validated against the Brief Symptom Inventory, the PHQ-4 has a specificity of 94.5% and sensitivity of 51.6% (22).

#### Further Questions

In addition to the validated questionnaires (IMET and PHQ-4), the health-related quality of life and subjective health status of the last 2 weeks for each participant were each assessed with a single item on a seven-point Likert-scale. Higher scores on the Likert-scale indicate a poorer health status or lower quality of life.

#### Measured Covariates

We obtained additional items in our questionnaire about sociodemographic variables including age (numeric and categorized), school education, gender, size of residential place and variables describing the living situation of the participants (e.g., single parent). School education was classified as low (no or low secondary school diploma), medium (intermediate secondary school diploma) or high (college preparatory) based on the German secondary school graduation. In addition, the questionnaire was used to obtain information about medical conditions/treatments, such as the underlying disease of the immunosuppressed participants, the degree of disability according to German Social Law (categorized) and if the person paused his/her immunosuppressive medication prior to receiving the COVID-19 vaccine.

#### Time Points

The baseline questionnaire, including sociodemographic and medical data, was administered at enrolment. The IMET, PHQ-4, health-related quality of life and health status questions were repeated in a follow-up questionnaire 1 month after the participant's second COVID-19 vaccination shot.

### Statistical Analysis

For the statistical analysis, recruited participants were excluded if they (a) did not state their immunosuppressive medication or underlying disease, (b) had a complete COVID-19 immunization at baseline (14 days after two vaccinations or after one in case the COVID-19 Vaccine Janssen was used) or (c) baseline and follow-up questionnaire were filled out with a time gap <21 days.

Characteristics of the sample were reported descriptively. Reliability of the included questionnaires were assessed calculating Cronbach's alpha. Cronbach's alpha values ≥0.7 were interpreted as acceptable (23). Mean scores and differences between baseline scores and follow-up scores (1 month after COVID-19 vaccination) were reported and compared using a paired *t*-test. Differences were calculated subtracting the follow-up scores from the baseline scores. Thus, higher scores indicate worsening and lower scores an improvement of the outcome. All examined outcomes were approximately normally distributed.

The effect size Hedges g^*^ adjusted for small sample size was calculated. Values of 0.2, 0.5, and 0.8 are interpreted as a small, medium, and large effect size, respectively (24). The PHQ-4 measures overall psychological distress as well as anxiety and depression symptoms in two subscales of the instrument (21, 25). A sum score of ≥3 on either subscale or ≥6 on the whole scale is considered the cutoff point for identifying possible symptoms of clinical anxiety or depression. According to this instrument, each patient was classified as “clinically unremarkable” or having “possible anxiety,” “possible depression” or “possible mental health concerns” at baseline. At follow-up, the same instrument was used to detect and classify possible abnormalities concerning anxiety, depression and overall mental health. Any changes in the PHQ-4 classifications between baseline and follow-up were tested with the McNemar-test. An alpha level of 0.05 or less was considered to be statistically significant. We adjusted the alpha using the Bonferroni correction when subscales of the questionnaires were individually tested.

Participants that did not complete both the baseline and follow-up questionnaires were excluded from the analysis. Bivariate analysis was conducted between sociodemographic variables and the paired IMET differences, while reporting the mean difference of the baseline and follow-up IMET scores and the 95% confidence interval [CI] using t-distribution. Pearson correlation coefficients between the IMET and the different questionnaires were calculated. According to Cohen (26), a correlation coefficient of 0.1, 0.3, and 0.5 is interpreted as a small, moderate, and strong association between two variables. All statistical analyses were carried out using SPSS Version 28 (IBM, Armonk, NY) while R (ggplot2 package) was used to illustrate the results in figures.

## Results

### Sample Description

**Figure 1:**
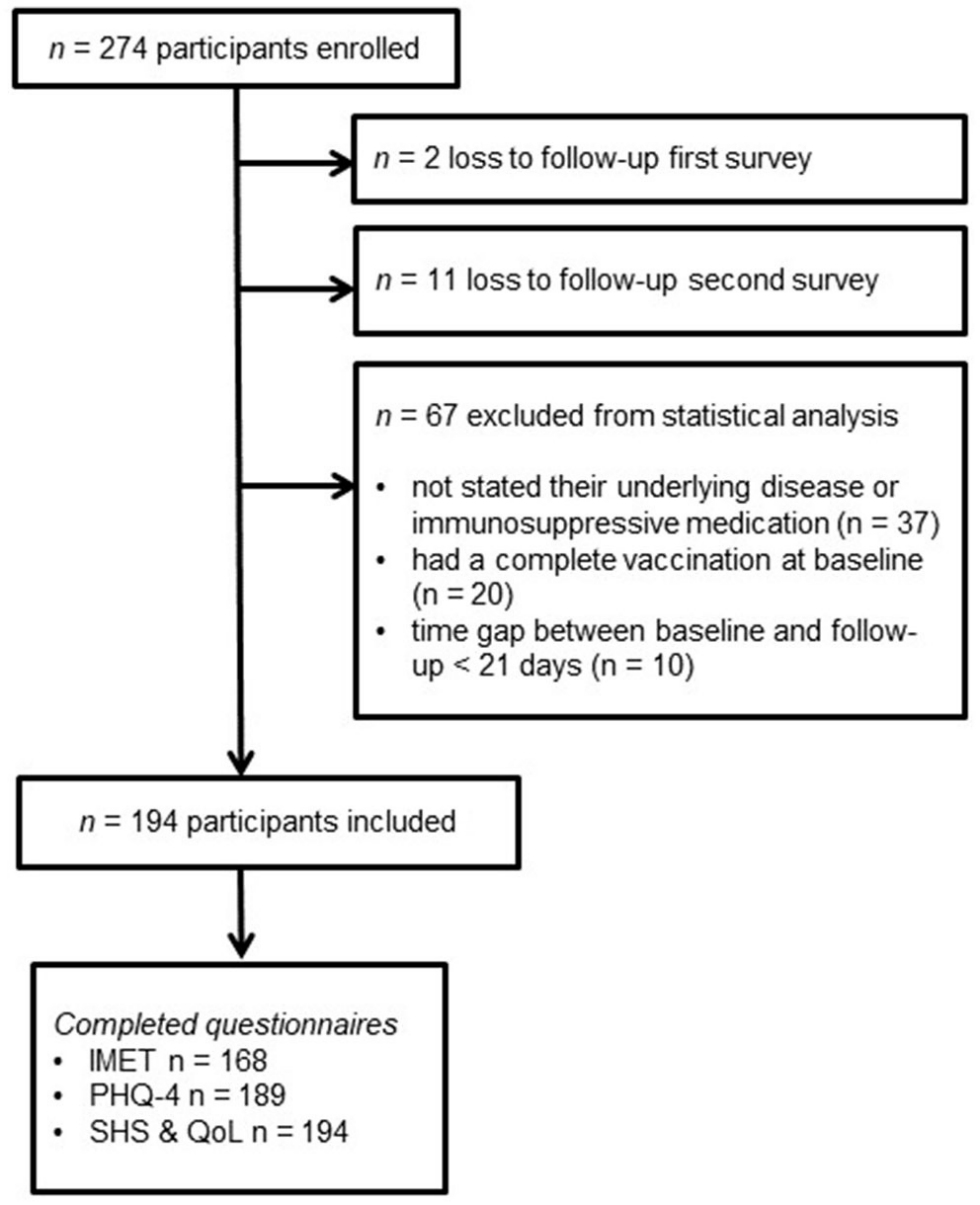
Flowchart participants' exclusion from analysis. Legend: IMET, Index for the Assessment of Health Impairments; PHQ-4, Patient Health Questionnaire-4; QoL, Quality of Life; SHS, Subjective health status.

The baseline questionnaire was filled out between March 30 and May 21, 2021. Between May 17, 2021, and August 30, 2021, the follow-up questionnaire was completed. The mean interval between the completion dates was 79.9 days (SD: 23.5, min: 23, max: 143). After loss-to-follow up and further exclusion of the participants based on the inclusion and exclusion criteria, a total of 194 participants were included in the analysis (see Figure 1). No participant completed the survey during the period of the national lockdown in Germany. The participants were on average 51.3 years old and mostly female (70.5%). The majority of our sample had a high school education (60.4%) and lived either in rural areas (<5.000 residents) or big cities (>100.000 residents). The most frequent diagnosis groups of the underlying immunosuppressive therapy were rheumatic diseases (*n* = 82, 42.3%), inflammatory bowel diseases (*n* = 39, 20.1%), and/or psoriasis (*n* = 27, 13.9%). About one third of the participants suffer from hypertension (*n* = 76, 39.2%). Further comorbidities are diabetes type 2 (*n* = 8, 4.1%), heart failure (*n* = 2, 1.0%) and COPD (*n* = 2, 1.0%). One quarter of all participants paused their immunosuppression medication due to the COVID-19 vaccine (Table 1). The IMET was completely covered at both time points by 168, PHQ-4 by 189 and quality of life and health status by all 194 participants, respectively. The reliability of the baseline and follow-up IMET and PHQ-4 questionnaires indicates a high internal consistency (Cronbach's alpha ≥0.8).

**Table 1:** Baseline characteristics.

	***n* (%) or mean (sd)**
**Gender**	
Male	57 (29.5)
Female	136 (70.5)
**Age, years (mean (sd))**	51.3 (13.8)
<40	44 (22.8)
40–65	115 (59.6)
>65	34 (17.6)
**School education** ^**1**^	
Low	16 (8.6)
Middle	54 (28.9)
High	113 (60.4)
Not specified	4 (2.1)
**City resident size**	
<5,000	77 (41.0)
5,000–20,000	36 (19.1)
20,000–100,000	24 (12.8)
>100,000	51 (27.1)
Household*	
Parenting	47 (24.2)
Single parent	2 (1.0)
Living alone	38 (19.6)
Care of relatives	22 (11.3)
Underlying disease*	
Rheumatological disease	82 (42.3)
Inflammatory bowel disease	39 (20.1)
Psoriasis	27 (13.9)
Multiple sclerosis	21 (10.8)
Transplant	14 (7.2)
Other	22 (11.3)
Comorbidities*	
Hypertension	76 (39.2)
Heart failure	2 (1.0)
Diabetes type 2	8 (4.1)
COPD	2 (1.0)
**Degree of impairment (%)** ^**2**^	
No impairment (0)	71 (36.8)
Low impairment (20–49)	39 (20.2)
Moderate impairment (50–74)	63 (32.6)
Severe impairment (75–100)	20 (10.4)
**Therapy paused for COVID vaccination (yes)**	48 (24.7)
Immunosuppression medication*	
Prednisolone	68 (35.1)
Methotrexate	52 (26.8)
TNF inhibitor	43 (22.2)
Azathioprine	13 (6.7)
Tacrolimus & Everolimus	12 (6.2)
Others	51 (26.3)
**Number of taken immunosuppressants**	
1	115 (59.3)
2	60 (30.9)
3 or more	19 (9.8)
**Vaccination type**	
mRNA	146 (77.7)
Vector-based	14 (7.4)
Cross vaccinated^3^	28 (14.9)

^1^based on German secondary school education, ^2^based on the German social law measuring physical, mental and social impairment ^*^Multiple selection possible; COPD: chronic obstructive pulmonary disease ^3^consisting of one dose mRNA vaccination and one dose vector-based vaccination.

### Change of Social Participation and Mental Health Over Time

At baseline, immunosuppressed participants had a mean IMET score of 31.7 compared to 27.2 at follow-up (t167 = 3.75, *p* < 0.001). Three out of nine domains of the IMET showed a significant change:(1) recreation and leisure, (2) social activities and (3) close personal relationships. The scores decreased between baseline and follow-up by 1.4 (recreation), 2.2 (social activities) and 0.7 (personal relationships), respectively (Table 2). The PHQ-4 with its subscales as well as the subjective health status and the quality of life showed no significant change between the two time points with effect sizes (Table 2).

**Table 2:** Mean characteristics of the measures and the effect size.

	**Baseline** **Mean (sd)**	**Follow-up** **Mean (sd)**	**Difference** **Mean (sd)**	Hedges g* **Hedges G (95% CI)**
**IMET Score T0-T1 (all completed cases** ***n*** **=** **168)**	**31.7 (16.7)**	**27.2 (18.3)**	**4.6 (15.7)**	**0.3 (0.1; 0.4)**
Usual activities of daily life (*n* = 194)^5^	1.3 (2.0)	1.5 (2.2)	−0.2 (1.8)	−0.1 (−0.2; 0.0)
Family and domestic responsibilities (*n* = 191)^6^	2.1 (2.3)	2.3 (2.5)	−0.2 (2.0)	−0.1 (−0.2; 0.0)
Getting thing done outside of home (*n* = 192)^4^	3.1 (3.0)	2.6 (2.7)	0.5 (2.9)	0.2 (0.0; 0.3)
Daily tasks and obligations (*n* = 191)^8^	2.8 (2.8)	2.6 (2.5)	0.2 (2.8)	0.1 (−0.1; 0.2)
**Recreation and leisure (*****n*** **=** **187)**^**9**^	**5.5 (3.4)**	**4.0 (3.1)**	**1.4 (3.6)**	**0.4 (0.2; 0.5)**
**Social activities (*****n*** **=** **188)**^**9**^	**7.1 (3.4)**	**4.8 (3.2)**	**2.2 (3.8)**	**0.6 (0.4; 0.7)**
**Close personal relationships (*****n*** **=** **194)**^**7**^	**3.6 (3.2)**	**2.9 (2.9)**	**0.7 (3.1)**	**0.2 (0.1; 0.4)**
Sex life (*n* = 185)^7^	3.2 (3.2)	3.6 (3.5)	−0.4 (3.0)	−0.2 (−0.3; 0.0)
Stress and extraordinary strain (*n* = 194)^2^	3.5 (2.8)	3.3 (2.8)	0.2 (2.7)	−0.1 (−0.1; 0.2)
PHQ-4 (*n* = 189)	2.9 (2.6)	2.8 (2.4)	0.1 (2.3)	0.0 (−0.1; 0.2)
PHQ-2 (*n* = 189)	1.6 (1.4)	1.5 (1.3)	0.1 (1.3)	0.1 (−0.0; 0.2)
GAD-2 (*n* = 191)	1.3 (1.5)	1.4 (1.4)	−0.0 (1.4)	−0.0 (−0.2; 0.1)
Subjective health status (*n* = 194)	3.2 (1.1)	3.2 (1.3)	0.0 (1.2)	0.0 (−0.1; 0.2)
Quality of Life (*n* = 194)	3.2 (1.3)	3.0 (1.2)	0.2 (1.3)	0.1 (−0.0; 0.3)

**Bold**: significant change between baseline and follow-up between using a paired t-test (p < 0.05 or adjusted after Bonferroni while testing subscales); Hedges g^*^ bias corrected for small sample size; sd: standard deviation; IMET, Index for the Assessment of Health Impairments; PHQ-4, Patient Health Questionnaire-4; PHQ-2, Patient Health Questionnaire-2; GAD-2, Generalized Anxiety Disorder Scale-2; ^superscript^ indicating targeted ICF domain.

The proportion of participants with questionnaire scores indicating mental health problems showed a slight but non-significant decrease between baseline and follow-up.

### Correlation Between Change in Social Participation and Other Measures

The difference between baseline and follow-up of the IMET showed a significant correlation with the difference of the PHQ-4, whereby the subscale PHQ-2 showed a small significant correlation and the correlation with the GAD-2 was not significant. A small significant correlation was also found between the self-rated quality of life and the IMET (Table 3). There was no difference regarding the type of vaccination (mRNA, vector-based vaccination and cross vaccination) between those participants whose social participation improved and those whose social participation stayed consistent or worsened.

**Table 3:** Correlation between the IMET differences and other subscales.

**Scale**	**Correlation (95% CI)**
**PHQ-4**	**0.34 (0.20–0.47)**
GAD-2	0.11 (−0.05–0.26)
**PHQ-2**	**0.26 (0.11–0.40)**
Subjective health status	0.13 (−0.02–0.27)
**Quality of life**	**0.29 (0.14–0.42)**

***Bold:** significant (Bonferroni-adjusted); PHQ-4, Patient Health Questionnaire-4; PHQ-2, Patient Health Questionnaire-2; GAD-2, Generalized Anxiety Disorder Scale-2*.

### Bivariate Analysis of Social Participation and Mental Health Across Sociodemographic Factors

**Figure 2:**
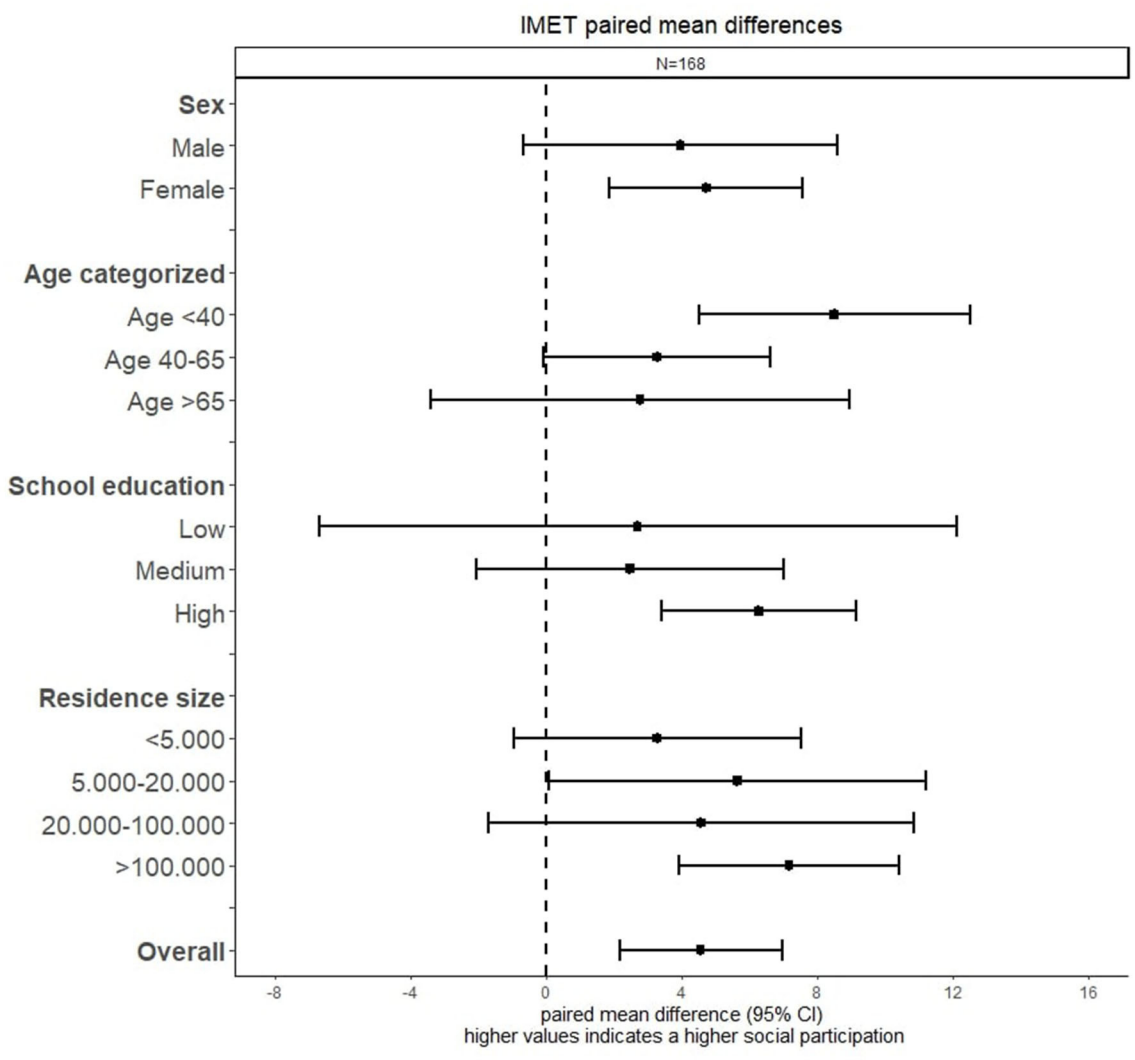
Forest plot of the IMET paired mean difference across sociodemographic factors.

Bivariate analysis of the IMET differences examined across social demographic variables shows overlapping 95% CI across all variables which indicates no significant differences using the t-distribution (Figure 2). Female participants (4.0, 95% CI [−0.7–8.6]) show a higher IMET difference compared to male participants (4.7, 95% CI [1.9–7.6]). With increasing age, a lower IMET difference can be observed. Participants with a low (2.7, 95% CI [−6.7–12.5]) or medium (2.5, 95% CI [−2.1–7.0]) school education had a nearly identical IMET score difference between baseline and follow-up, where participants with a high school education are associated with higher mean difference (6.3, 95% CI [3.4–8.9]). With increasing residential size, no clear pattern could be observed. However, participants living in larger cities with 100.000+ residents showed the highest IMET score difference (7.2, 95% CI [3.9–10.4]) between the two measured time points.

**Figure 3:**
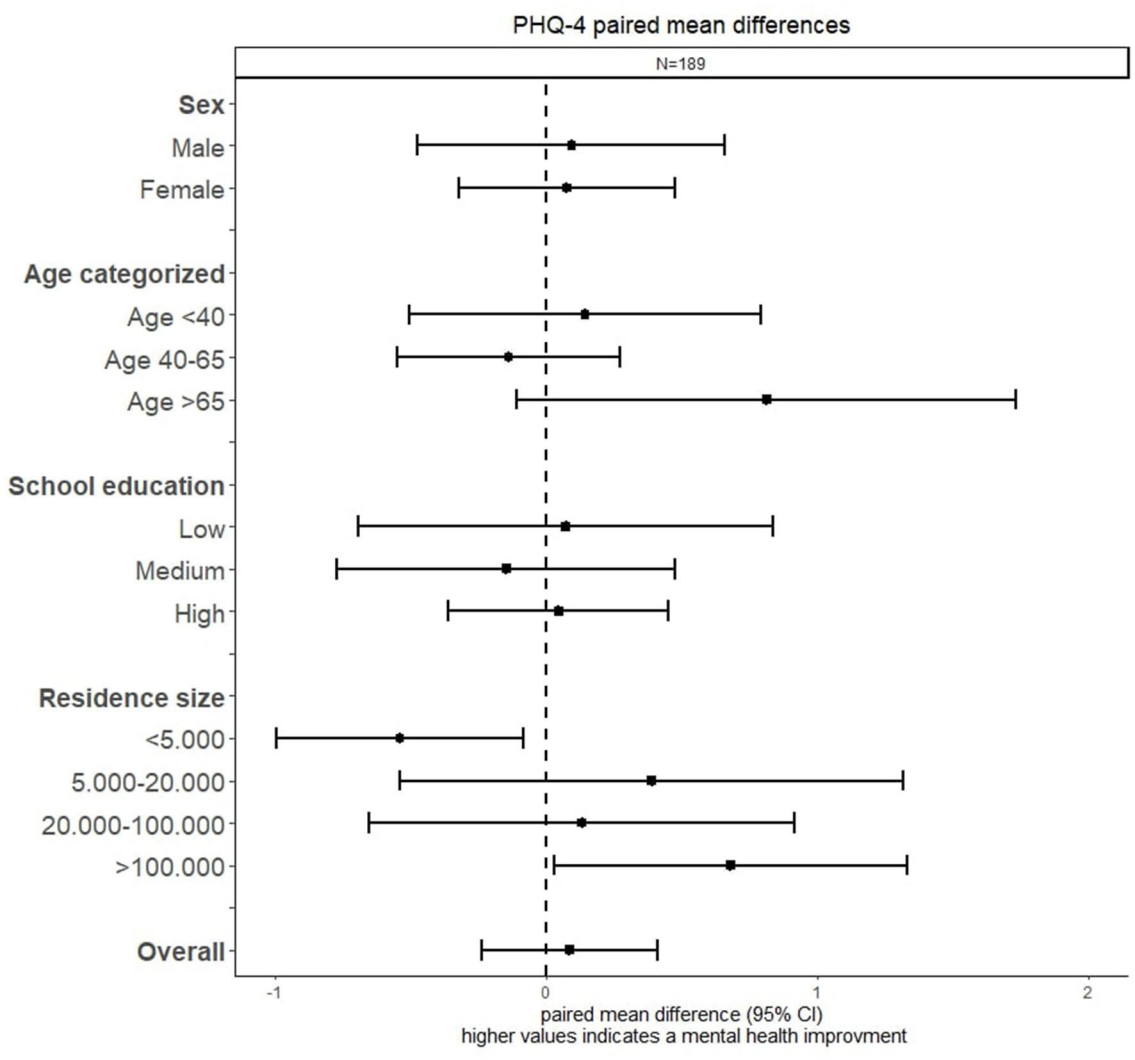
Forest plot of the PHQ-4 paired mean difference across sociodemographic factors.

While the mental status measured by PHQ-4 did not change significantly in the overall cohort, there was a significant difference between residents of villages and residents of large cities. For the first, the PHQ-4 scores worsened significantly (−0.5, 95% CI [−1.0 – −0.1]), while for residents of large cities the score improved considerably (0.7, 95% CI [0.0–1.3]) (Figure 3).

## Discussion

This study shows that social participation increased after complete COVID-19 vaccination. Three subscales “recreation and leisure,” “social activities” and “close relationships” were responsible for the change in the overall IMET score, while other aspects like “usual activity of daily life” remained the same. COVID-19 vaccination did not have an influence on mental health, subjective health status and quality of life. A positive and significant correlation was found between social participation and mental health status and between social participation and quality of life. No change of social participation was observed when stratified according to sociodemographic factors. Mental health did not differ significantly between baseline and follow-up, however, the subgroups of participants residing in small cities or villages (<5,000 residents) had a significant decrease of their mental health at follow-up and participants from big cities (>100,000 residents) showed a significant increase.

Even though the IMET was not initially developed to measure social participation during a pandemic, it was already used by Mergel & Schützwohl for this purpose (2021). They used the IMET to measure the social participation before and after the national lockdown during the COVID-19 pandemic. The study included participants from the general population as well as participants who suffered from a chronic mental disorder or participants who received active mental disorder treatment (20). The first observation point was before the pandemic began in August 2019 followed by March 2020 and July 2020. Similar to our results, a lower social participation in all groups in the subdomains recreation and leisure, social activities and close personal relationships was observed at follow-up. These domains were presumably mostly directly affected by public health measures implemented to contain the pandemic.

The change of the IMET can be compared with already existing literature using norm data, pre-post changes and intergroup pre-post changes from rehabilitation research. Deck et al. (8) gathered norm data for the IMET in the German population in 2014. A mean IMET score of 16.65 for women and 15.6 for men aged between 50 and 59 years was described. People with chronic inflammatory bowel disease had an IMET score about 18.7. Our sample, with a mean age of 51.3 years, had considerably higher mean IMET scores of 31.7 at baseline and 27.2 at follow-up, indicating less social participation. However, the pandemic situation and an underlying disease that requires immunosuppressive medication were key differences between the norm data and our sample. Furthermore the baseline characteristics also differed from the German average population, especially the comparatively high level of education in our sample as well as the higher proportion of female participants (8). Hueppe et al. (27) compared in a randomized controlled trial the effect of a rehabilitation intervention in participants with inflammatory bowel disease. The control group and the intervention group showed IMET scores of 32.8 and 34.7, respectively. The IMET score decreased by 7.3 and 2.9 points in the intervention and control groups, respectively, after 12 months. These changes from baseline to follow-up resulted in an effect size between the study arms of 0.23. In our sample, with 20% diagnosed inflammatory bowel disease, vaccination of immunosuppressed participants resulted in an even higher effect size (0.29). A similar effect size of 0.36 was found by Nübling et al. studying a rehabilitation intervention using secondary data of participants with a mean age of 51.7 years (28). Comparing the effect sizes of our study with rehabilitation interventions, we found the effect of the complete COVID-19 vaccination and the effect of a rehabilitation intervention to be comparable.

In contrast to our finding of no significant change of mental health between the two time points, a study among hospital workers found a significant difference in the rate of change in vaccinated persons compared to non-vaccinated persons implying better mental health after the COVID-19 vaccination (29). The correlation between increasing IMET and increasing PHQ-2 scores leads to the assumption that a high level of depressions goes along with less social participation. The absence of a correlation between the change in IMET and GAD-2 between baseline and follow-up shows no dependency between social participation and anxiety levels in our sample. Further studies confirm the association between social participation and depressive symptoms. For example Noguchi et al. could show that effect as well during the COVID-19 pandemic (12, 30). The difference in mental health between participants living in small cities or villages and big cities may be explained due to lower mental health care resources in rural areas compared to big cities (31). This finding needs to be investigated further in a multivariable model adjusted for various confounders with a larger sample size and over a longer period of time. The vaccination type showed no association to the change of social participation. It has to be considered that about 75% of our cohort were vaccinated with a mRNA vaccination.

The German National Institute for Public Health (“Robert Koch-Institut”) reports a reduced immune response and suggests a reduced effectiveness for immunocompromised persons (25). Even for healthy individuals, the effectiveness of vaccination cannot be assessed with certainty with regard to the virus variants of concern (32). Official recommendations for high-risk groups recommend severe and more far-reaching restrictions regarding lifestyle and protection measures compared to statutory restrictions (33). Further evidence regarding the immune response, efficacy and duration of protection of the COVID-19 vaccination for immunocompromised persons could have negative effect upon the social participation of these persons, even after the initial improvement.

There has never been a pandemic in the recent history of time. Existing scientific concepts, e.g., for measuring participation, cannot cover the dimensions of impairment. The IMET used as a primary outcome in the study is based on the International Classification of Functioning, Disability and Health and developed to measure the effect of rehabilitation interventions and may not reflect the social participation completely during a pandemic. The data from this study could be used for sample size calculation in further research. Based on the IMET score change of 4.6 and a standard deviation of 15.6 after a complete vaccination a sample size of 94 would be sufficient to detect such an effect with a power of 80% and alpha 0.05 using a paired *t*-test.

Various factors beside the vaccination status could confound our results. Possible confounders could be changes regarding the pandemic situation, disease progression and medical therapy. In particular, the incidence of SARS-CoV-2 infections varies over time, with a trend toward higher incidence at colder outdoor temperatures. SARS-CoV-2 incidence and the proportion of intensive care unit beds occupied by COVID-19 patients to the total number of intensive care unit beds were used as the main reference values for regulatory restrictions to minimize the spread of SARS-CoV-2 (34). A further limitation of the study is that the results may be influenced by changes in the season (from spring to summer), which may have an effect upon a respondent's perception of reduced social participation. Further studies should compare social participation during the different seasons of the year in immunocompromised persons. They are more vulnerable for any kind of contagious disease, not just COVID-19, and seasonal waves of illness may have an effect upon social participation.

The sample of our study may not be representative for all immunocompromised persons (e.g., mostly female and low comorbidities) due to possible selection bias and loss-to-follow-up bias. Only complete cases for each included scale were analyzed. Therefore, the number of participants varies between included outcomes.

Additionally, only immunocompromised persons who wanted to get vaccinated were recruited. Therefore, the results are based on paired differences between two time points. A study design including a non-vaccinated, immunocompromised control group would have allowed us to estimate the effect of the vaccination on the social participation with more validity. However, a study design requiring immunocompromised persons to remain unvaccinated over the 12-month study period would neither have been feasible nor ethically appropriate.

By offering only questionnaires in German language, we structurally excluded potential participants with limited German language proficiency. The main cause of this was that the IMET questionnaire is only validated in the German language.

Participants could have misunderstood the items of the questionnaire even though they could contact the research team and ask questions about the individual items.

## Conclusion

The investigation of immunocompromised participants revealed a positive change in social participation after a complete COVID-19 vaccination. The improvement of participation after vaccination corresponds in effect size to that of medical rehabilitation. An increase of social participation was observed in the domains “recreation and leisure,” “social activities” and “close personal relationships.” Social participation was positively associated with mental health and quality of life in our sample. Across different sociodemographic factors, no differences in social participation were observed. The dynamic pandemic situation could influence social participation additionally to vaccination status. The hypothesis that social participation is positively affected by complete COVID-19 vaccination should be examined in further studies, including a control group where possible to ensure these results.

## Data Availability Statement

The raw data supporting the conclusions of this article will be made available by the authors, without undue reservation.

## Ethics Statement

The studies involving human participants were reviewed and approved by Ethics Committee of the University Medical Center Göttingen (No. 29/3/21). The patients/participants provided their written informed consent to participate in this study.

## Author Contributions

Conceptualization: GH and FM. Methodology: DS and GH. Formal Analysis: DS. Investigation: GH, SH, and FM. Writing – original draft preparation: GH and DS. Writing – review and editing: SH, FM, MM, SS, AD-J, GB, EH, FK, JN, MM, and AC. Visualization: DS. Funding acquisition: FM, AD-J, SH, and EH. All authors have read and agreed to the published version of the manuscript.

## Funding

Parts of the study were performed within the DEFEAT Corona project, funded by the European Fund for Regional Development (EFRD) (Funding No: ZW7-85152953). The funding source had no role in the studies' design, execution, analyses, interpretation of the data or decision to submit the results.

## Conflict of Interest

The authors declare that the research was conducted in the absence of any commercial or financial relationships that could be construed as a potential conflict of interest.

## Publisher's Note

All claims expressed in this article are solely those of the authors and do not necessarily represent those of their affiliated organizations, or those of the publisher, the editors and the reviewers. Any product that may be evaluated in this article, or claim that may be made by its manufacturer, is not guaranteed or endorsed by the publisher.

## Abbreviations

GAD: Generalized Anxiety Disorder Scale
IMET: Index for the Assessment of Health Impairments
PHQ Patient Health Questionnaire.
